# Supplementary material for: Responsiveness and interpretability of the pain subscale of the Knee and Hip Osteoarthritis Outcome Scale (KOOS and HOOS) in osteoarthritis patients according to COSMIN guidelines
Source: PLoS One. 2023 Nov 16;18(11):e0293760. doi: 10.1371/journal.pone.0293760 (PMC10653527; doi:10.1371/journal.pone.0293760)
Supplement: Attachment — Submitted filename: Response to Reviewers.docx [file pone.0293760.s002.docx]

Dear Editor, 

Thank you for considering our manuscript for publication. We appreciate the time and effort the reviewers took to review the manuscript so extensively.

Below, a point-by-point response is provided to all the peer reviewers’ comments and the comments from the Academic Editor. Also, the additional requirements of the journal are addressed below.

We hope that with these revisions, the manuscript will fully meet PLOS ONE’s publication criteria.

Kind regards on behalf of all the authors,

Wietske Rienstra

| **Comments by the academic editor:** | **Author’s response** |
| --- | --- |
| Line 16: “…osteoarthritis patients and after Total Knee Arthroplasty and Total Hip Arthroplasty (TKA/THA).”  should be corrected to “…osteoarthritis patients and evaluation of the results after Total Knee Arthroplasty (TKA) and Total Hip Arthroplasty (THA).” | done |
| The authors should give a small hint about the COSMIN guidelines in the “background” section in the “Abstract” (1-2 sentences) | See the additional sentences in the manuscript (Line 21-24) |
| Lines 21-22:  “This responsiveness study was conducted using data obtained from the DOA trial  (Duloxetine in OsteoArthritis).”  should be corrected to ““This responsiveness study was conducted using data obtained from the Duloxetine in OsteoArthritis (DOA) trial.” (the same in Lines 93 and 94) | done |
| Line 35: “93 participants” should be corrected to ‘Ninety- three participants” | done |
| Lines 36-37-39: what is the meaning of “resp.” | The meaning is ‘respectively’. For clarification I corrected the abbreviation into the complete word in the manuscript |
| Line 67 “Total Knee Arthroplasty and Total Hip Arthroplasty (TKA/THA)” better to be changed to “Total Knee Arthroplasty (TKA) and Total Hip Arthroplasty (THA) | Thank you for this suggestion, see the correction in the manuscript |
| Line 90: “…subscales in patients with knee or hip OA as well as patients after TKA/THA” better to be changed to ““…subscales in patients with knee or hip OA as well as patients after TKA and THA” (the same for line 96) | Thank you for this suggestion, TKA/THA was changed into TKA and THA throughout the entire manuscript |
| Line 116: “HOOS/KOOS” should be corrected to Knee and Hip Osteoarthritis Outcome Scores (KOOS and HOOS) | done |
| HOOS/KOOS | Changed into KOOS and HOOS throughout the entire manuscript |
| Lines 125-130: “Collins et al. report  that the Pain subscale especially, and also the ADL subscale, and QOL subscale of the KOOS show  the greatest relevance, room for improvement, and effect sizes in knee OA patients undergoing  TKA. Collins et al. report a Smallest Detectable Change (SDC) of 26 points on individual level for  the pain subscale of the KOOS in patients with OA[3]. To our knowledge, similar information is  lacking in literature regarding the HOOS.” These sentences are not a “Methodology” and should be moved to the “Discussion” section | This paragraph was moved to the discussion section |
| Lines 142-144: “The Dutch version of the mPDQ is considered to be a fairly reliable and valid self-report  instrument in patients with hip and knee OA with an individual SDC of 7.3 points for hip/knee OA  patients[17,18]. This sentence is not a “Methodology” and should be moved to the “Discussion” section | This paragraph was moved to the discussion section |
| Line 150: “VAS have been reported as valid and reliable measures for the intensity of pain” this sentence should be deleted | This sentence was deleted |
| Line 248: The meaning of the abbreviation “BMI” was not previously mentioned. | The abbreviation was clarified |
| **Comments by Reviewer #1:** | **Author’s response** |
| Line 21 and line 93: Can the authors please explain what they mean by “responsiveness study”. Further clarity on this study design is needed as this is not clear in the manuscript and no evidence to support it was found in the literature. If this is an oversight, authors should kindly indicate the appropriate study design. | Thank you for this comment. The study design was clarified into: clinimetric study |
| Line 245: Please consider reviewing this sentence as the phrase (see chapter 5 of this thesis) is out of place. | this sentence was reviewed. The mentioned chapter 5 has been changed into the proper reference (the paper has been published) |
| Line 253: A bit difficult to understand the table. Perhaps authors will consider repositioning the headers “Baseline”, “After conservative treatment” and “Six months after TKA/THA” for better presentation. | the presentation of the table was changed with the aim of improving readability |
| **Comments by Reviewer #2:** | **Author’s response** |
| The overall perception of patient’s condition can be captured in a simple way with use of different instruments containing anchor questions. Why have you chosen the Global Impression Scale of Improvement? | Thank you kindly for this suggestion. In the period in which the trial was conceived we were unaware of such instruments. Therefore we used the Global Impression Scale of Improvement as an anchor. |
| The differences in the mean score changes in the Pain subscales between patients undergoing knee and hip arthroplasties results from the different time of recovery following these operations. It should be mentioned in the Discussion section. | Thank you for this very true comment. However, since a clinimetric study as this regards whether the measured change scores are valid rather than the magnitude of the changes (or the true difference between hip and knee arthroplasty patients), this comment is more relevant for discussion in the paper regarding the primary trial results (Rienstra et al. 2021. BMJ Open). Therefore we chose to discard this comment in order to keep the clinimetric character of this paper intact. |
| In the Results section you mentioned that “reasons for loss to follow-up were described previously (…)” (line 245). It has not been done. As I understand this sentence was copied from the thesis (“see chapter 5 of this thesis”), and is not consistent with the present manuscript. Please, correct it. | See also the reply to reviewer #1 regarding this issue. The appropriate reference was added. |
| I would however like to see the data concerning loss to follow-up. This feature was not presented or discussed in your manuscript. | As this information is provided in a free access paper we chose not to elaborate on this part for multiple reasons:   1. To avoid repeating of identical results and information in different papers 2. Because we think that the context of this information is better described and more relevant in the paper regarding the original trial 3. To improve readability of the present manuscript. |

Journal Requirements:

When submitting your revision, we need you to address these additional requirements.

1. Please ensure that your manuscript meets PLOS ONE's style requirements, including those for file naming. The PLOS ONE style templates can be found at

<https://journals.plos.org/plosone/s/file?id=wjVg/PLOSOne_formatting_sample_main_body.pdf>and

<https://journals.plos.org/plosone/s/file?id=ba62/PLOSOne_formatting_sample_title_authors_affiliations.pdf>

**We have updated the style according to the abovementioned requirements.**

2. In your Data Availability statement, you have not specified where the minimal data set underlying the results described in your manuscript can be found. PLOS defines a study's minimal data set as the underlying data used to reach the conclusions drawn in the manuscript and any additional data required to replicate the reported study findings in their entirety. All PLOS journals require that the minimal data set be made fully available. For more information about our data policy, please see <http://journals.plos.org/plosone/s/data-availability>.

Upon re-submitting your revised manuscript, please upload your study’s minimal underlying data set as either Supporting Information files or to a stable, public repository and include the relevant URLs, DOIs, or accession numbers within your revised cover letter. For a list of acceptable repositories, please see <http://journals.plos.org/plosone/s/data-availability#loc-recommended-repositories>. Any potentially identifying patient information must be fully anonymized.

Important: If there are ethical or legal restrictions to sharing your data publicly, please explain these restrictions in detail. Please see our guidelines for more information on what we consider unacceptable restrictions to publicly sharing data: <http://journals.plos.org/plosone/s/data-availability#loc-unacceptable-data-access-restrictions>. Note that it is not acceptable for the authors to be the sole named individuals responsible for ensuring data access.

We will update your Data Availability statement to reflect the information you provide in your cover letter.

**We have included the dataset underlying the results as a supplementary file (corrected the file name to make it clearer). The location of the underlying dataset in the supplementary file is mentioned in line 299.**

3. Please include captions for your Supporting Information files at the end of your manuscript, and update any in-text citations to match accordingly. Please see our Supporting Information guidelines for more information: <http://journals.plos.org/plosone/s/supporting-information>.

**We have included these.**

4. Please review your reference list to ensure that it is complete and correct. If you have cited papers that have been retracted, please include the rationale for doing so in the manuscript text, or remove these references and replace them with relevant current references. Any changes to the reference list should be mentioned in the rebuttal letter that accompanies your revised manuscript. If you need to cite a retracted article, indicate the article’s retracted status in the References list and also include a citation and full reference for the retraction notice.

**The reference list was updated**
